# Genetic factors, risk prediction and AI application of thrombotic diseases

**DOI:** 10.1186/s40164-024-00555-x

**Published:** 2024-08-27

**Authors:** Rong Wang, Liang V. Tang, Yu Hu

**Affiliations:** 1 Institute of Hematology, Union Hospital, Tongji Medical College, Huazhong University of Science and Technology, Wuhan, China; 2 Key Lab of Molecular Biological Targeted Therapies of the Ministry of Education, Union Hospital, Tongji Medical College, Huazhong University of Science and Technology, Wuhan, China

**Keywords:** Thrombophilia, Genetic factors, Risk prediction, Artificial intelligence, Machine learning

## Abstract

In thrombotic diseases, coagulation, anticoagulation, and fibrinolysis are three key physiological processes that interact to maintain blood in an appropriate state within blood vessels. When these processes become imbalanced, such as excessive coagulation or reduced anticoagulant function, it can lead to the formation of blood clots. Genetic factors play a significant role in the onset of thrombotic diseases and exhibit regional and ethnic variations. The decision of whether to initiate prophylactic anticoagulant therapy is a matter that clinicians must carefully consider, leading to the development of various thrombotic risk assessment scales in clinical practice. Given the considerable heterogeneity in clinical diagnosis and treatment, researchers are exploring the application of artificial intelligence in medicine, including disease prediction, diagnosis, treatment, prevention, and patient management. This paper reviews the research progress on various genetic factors involved in thrombotic diseases, analyzes the advantages and disadvantages of commonly used thrombotic risk assessment scales and the characteristics of ideal scoring scales, and explores the application of artificial intelligence in the medical field, along with its future prospects.

## Introduction

**Fig. 1:**
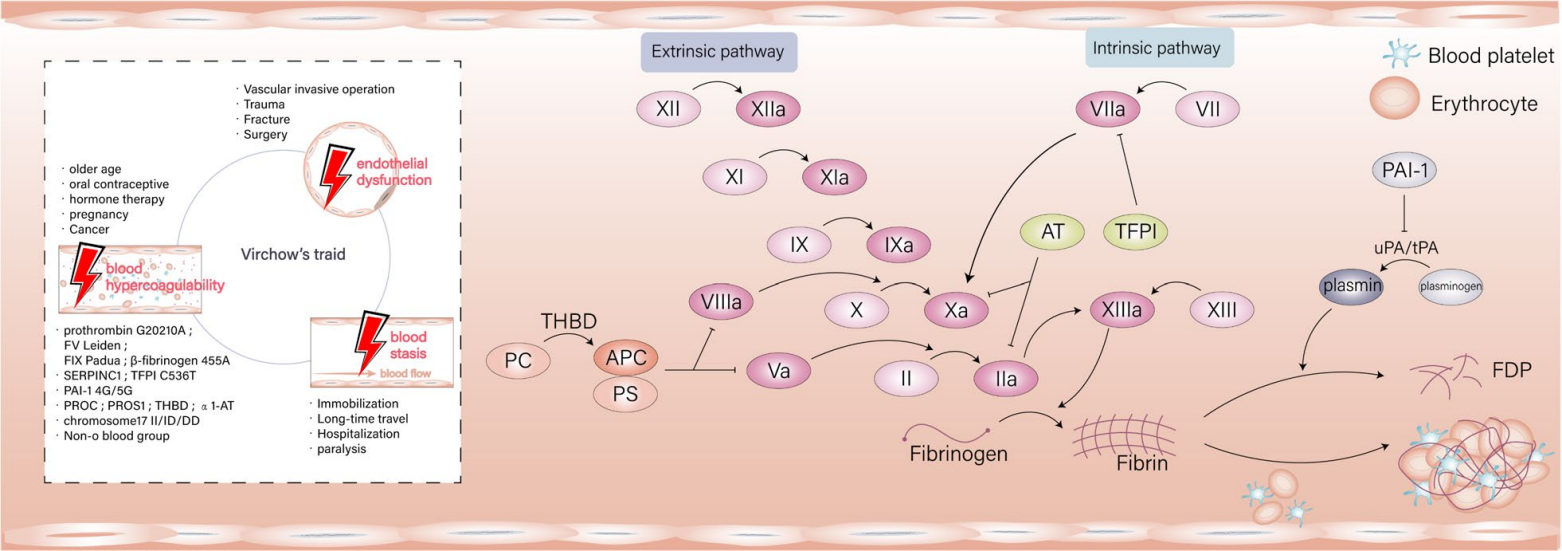
The Virchow’s triad and the coagulant-anticoagulant-fibrinolytic system. The Virchow triad consists of vascular endothelial dysfunction, blood hypercoagulability and blood stasis. The risk factors of thrombotic diseases are divided into two categories: acquired and hereditary, as shown above. Hereditary factors mainly lead to hypercoagulable state. The balance of coagulation, anticoagulation and fibrinolysis makes the blood flow smoothly in the blood vessel and has the potential ability of thrombosis. Changes in the antigenic level or activity of any substance in this balance are likely to break the balance and lead to thrombosis

Thrombotic disease is a systemic condition that encompasses venous thrombosis, arterial thrombosis, and intracardiac thrombosis. Venous thromboembolism (VTE) can be categorized as deep vein thrombosis (DVT) and pulmonary embolism (PE). Vascular endothelial cells secrete coagulation, anticoagulation, and fibrinolysis substances, along with corresponding regulatory molecules, which are tightly regulated in the blood vessels. Maintaining this delicate balance ensures smooth blood flow and preserves clotting potential. Acquired risk factors for thrombosis include certain medications, venous catheterization, surgery, prolonged immobilization, advanced age, and malignancies. Disruption of this balance by these factors can induce a hypercoagulable state, endothelial injury, or blood stasis, thereby increasing the risk of thrombosis (shown in Fig. 1). Genetic elements also play pivotal roles in disrupting this balance and promoting abnormal coagulation processes. Patients with a family history of thrombosis may opt for genetic screening, as observed, 45% to 60% of VTE events are associated with prothrombotic genotypes [1]. However, the decision lies with the patients, as clinicians do not recommend or guide unnecessary tests for diagnosis and treatment. Common clinical thrombosis susceptibility genes primarily include mutations in coagulation factors, coagulation inhibitors, the protein C system, the fibrinolytic system, and other genes outside the blood system.

Thrombotic disease is considered the primary cause of mortality worldwide and can manifest as an independent condition or as a secondary complication of specific diseases. In clinical practice, individuals with risk factors for thrombosis undergo routine thrombosis risk assessment and stratification. The most frequently employed tool is Risk Assessment Models (RAMs), which incorporate patient symptoms, signs, and risk factors to assign corresponding scores. However, these scales are often developed based on population-specific data from a particular institution or region, limiting their generalizability. Additionally, subjective factors of clinicians may introduce heterogeneity in the practical application of these scales.

With the development of artificial intelligence (AI), a growing number of applications in the field of medicine and health have emerged. These applications include, but are not limited to, the establishment of risk assessment models, assisting clinical diagnosis, guiding clinical decision-making, drug target screening, and patient management. Computer vision imaging can enhance the diagnostic accuracy of medical images and reduce the occurrence of false positive and false negative results [2]. Natural language processing can facilitate the standardization of electronic medical records, enabling convenient data screening in multi-center clinical studies. The use of auxiliary robots improves the success rate of precision procedures. Machine learning algorithms, such as deep learning, decision trees, and neural networks, can extract meaningful variables from big data to construct clinical models [3–5]. While retrospective studies have demonstrated the superiority of models based on large-scale real-world data compared to those based solely on clinical data, they are still insufficient to support clinical translation. This article primarily focuses on different forms of hereditary thrombophilia mutations across various systems, including classical mutations and newly discovered mutation sites. Additionally, it introduces commonly used thrombus scoring scales in internal medicine, oncology, and surgical patients, analyzing and comparing their advantages and disadvantages. Finally, it discusses the current application of AI in thrombotic diseases, the challenges faced at this stage, and potential directions for future development.

## Genetic factors of thrombotic diseases

The physiological anticoagulant mechanisms in the body can be primarily categorized into three groups: serine protease inhibitors, the protein C system, and tissue factor pathway inhibitors. Any increase in the plasma antigen level or activity of coagulation factors, a lack of anticoagulant substances, or low levels of fibrinolytic enzymes can significantly increase the likelihood of thrombosis. Genetic factors related to thrombotic diseases exhibit regional and ethnic differences. Multiple meta-analyses have revealed that protein C, protein S, and antithrombin deficiency are the most common genetic mutations found in East Asian populations, while coagulation factor V and prothrombin mutations are the primary mutations in Caucasian populations [6, 7]. This disparity may result from the combined effects of race and region, providing a background and basis for the focus of genetic screening in different populations to improve the efficiency of genetic counseling. Performing genetic testing for suspected hereditary thrombophilia not only aids in evaluating the prognosis of patients with thrombosis but also facilitates the discovery of carriers within thrombophilia families.

### Abnormal activation of coagulation cascade

**Fig. 2:**
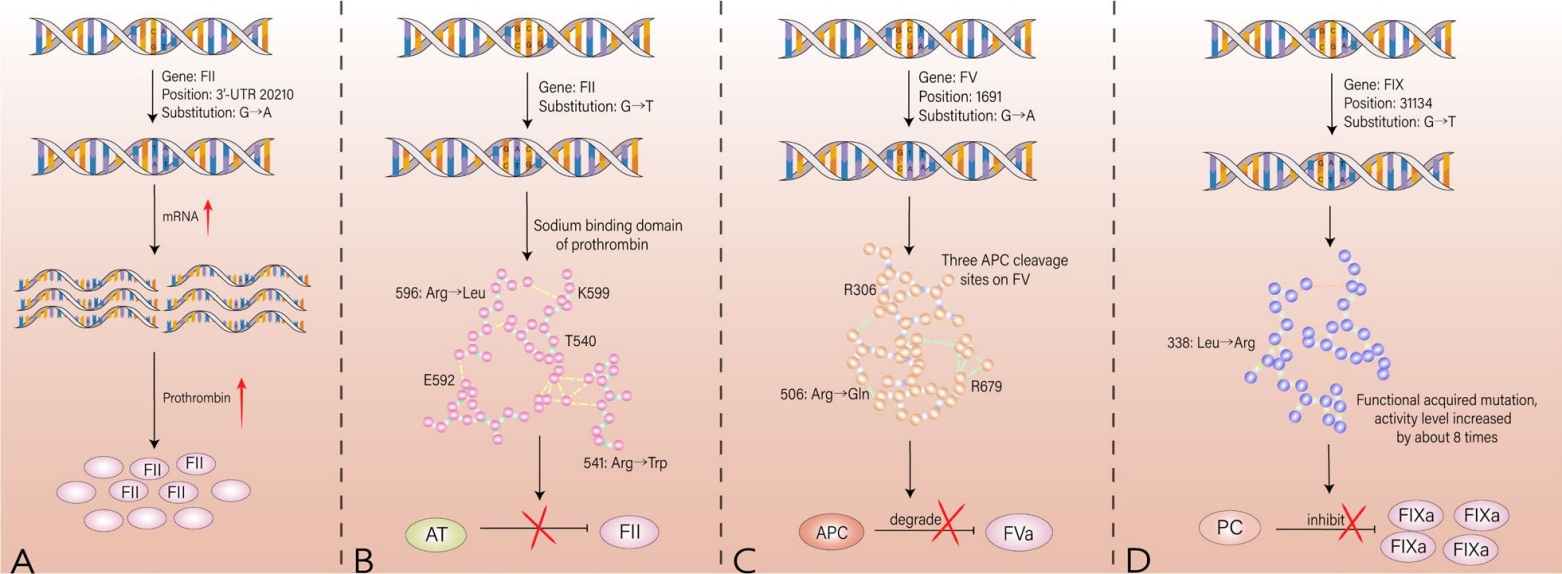
Coagulation factor mutations lead to thrombosis. **A** Most of the mutations in prothrombin are single nucleotide substitutions (G20210A) at 20210 in the 3 ' non-coding region, resulting in abnormal 3 ' -terminal cleavage signals, accumulation of transcribed RNA, and corresponding increase in prothrombin produced at the translation level. **B** Prothrombin is a sodium-regulated allosteric enzyme. The sodium-binding domain consists of five amino acid residues: Thr540, Arg541, Glu592, Arg596 and Lys599.Single nucleotide substitution caused by mutations in this site easily leads to antithrombin resistance. **C** Arg506 is a kinetically favorable APC cleavage site, and the missense mutation at position 1691 causes Arg to be replaced by Gln (FV Leiden). On the one hand, FVa continues to express procoagulant activity, on the other hand, it resists the cleavage of APC, causing APC resistance-related thrombosis events. **D** The FIX R338L mutation was a gain-of-function mutation (FIX Padua), and the plasma level of FIX was normal, while the activity level was increased by about 8 times. At the same time, the stability of the combination with PS decreased, and the inhibition of PS decreased, showing a clear tendency of thrombosis

Coagulation cascades can be classified into endogenous and exogenous pathways. Genetic mutations in various coagulation factors have been found to be associated with thrombotic events (shown in Fig. 2). Prothrombin, the zymogen form of thrombin, acts as a proteolytic enzyme that hydrolyzes multiple coagulation factors, promotes the coagulation cascade reaction, and converts soluble fibrinogen into a fibrin network, facilitating the cross-linking of red blood cells and platelets. Additionally, prothrombin can participate in the activation of protein C and exert anticoagulant activity.

Most prothrombin mutations are single nucleotide substitutions occurring at position 20210 in the 3' non-coding region. These mutations result in abnormal 3'-terminal cleavage signals, leading to an accumulation of transcribed RNA and an increase in prothrombin production at the translation level [8–10]. Co-transfection experiments have confirmed that these mutations do not affect the molecular structure but create a more efficient cleavage site compared to the wild type, thereby increasing the efficiency of 3' end processing [11]. Heterozygous mutations of prothrombin lead to an approximately 30% increase in plasma levels, while homozygous mutations result in around a 70% increase. Even at the same plasma level, carriers of the mutant alleles produce more thrombin and exhibit activated protein C (APC) resistance compared to non-carriers [12]. This mutation also enhances the activation of thrombin-activated fibrinolysis inhibitor in a concentration-dependent manner, resulting in prolonged plasma clot lysis time [13].

In a multicenter study on portal vein thrombosis [14], it was found that the frequency of cytomegalovirus (CMV) positive patients with the G20210A gene mutation is higher, indicating a potential synergistic effect on thrombosis. However, the temporal relationship between CMV infection and the occurrence of the gene mutation needs to be confirmed through prospective studies [15, 16]. Based on this finding, further investigations are warranted to determine whether similar associations exist when CMV is combined with other coagulation factor mutations.

Recent studies have made new discoveries in the pathogenesis of prothrombin gene mutation (PGM). Prothrombin is an allosteric enzyme that is regulated by sodium and has a sodium-binding domain consisting of five amino acid residues: Thr540, Arg541, Glu592, Arg596, and Lys599. Single nucleotide substitutions caused by mutations at this domain can lead to antithrombin resistance [17].

Arginine at position 596 is an important site for regulating thrombin activity. Replacing it with leucine may slightly reduce activity but greatly reduce antithrombin binding ability, leading to antithrombin resistance and increased susceptibility to thrombosis. Through genome-wide association analysis and whole-exome sequencing (WES), Mulder et al. [18] found that replacing the arginine residue at position 541 with tryptophan made prothrombin resistant to antithrombin. Building on this finding, Wu et al. [19] discovered that the mutation was accompanied by a decrease in prothrombin procoagulant activity. More significantly, the mutation impaired protein C (PC) activation, and the combined effect ultimately biased PGM towards procoagulant activity. Arg541Trp mainly impairs the binding affinity of PC, resulting in low anticoagulant efficiency of activated protein C (APC), but does not affect the interaction between thrombin and thrombomodulin. This finding suggests that beyond common hereditary PC, PS defects, and coagulation factor V Leiden mutations, inefficient PC pathway activation caused by prothrombin abnormalities may contribute to activation defects in the PC pathway. Although the mechanism of this effect has yet to be fully elucidated, this study sheds new light on the mechanism of thrombosis.

Nonsense mutations in the prothrombin gene can lead to the formation of prothrombotic phenotypes, in addition to single nucleotide substitutions. A CGC > CGT codon replacement (c.1824C > T) in the last exon of the prothrombin gene results in an unchanged protein product, but increased mRNA expression [20]. Computer prediction shows that there may be secondary structure recombination, prolonging half-life and increasing quantity. In vitro studies observed hyperprothrombinemia, hypofibrinolysis, and dense fibrin clots with impaired fibrinolytic function. However, routine hemostatic tests in the laboratory could not verify the procoagulant state because no correlation was observed between the increase in plasma concentration and activity. This separation between concentration and activity also exists in G20210A [21]. Prior to this report, synonymous single nucleotide polymorphisms were seldom linked to diseases, with amino acid sequences being considered clinically insignificant. This report reveals that changes in codons may affect the effectiveness of proteins in terms of quantity, structure, and function, despite the transcripts being the same. This provides new ideas for clinical diagnosis and treatment. When common genetic factor screening and laboratory tests are negative and there is no apparent cause of thrombosis, it is possible to consider whether it is caused by synonymous mutations.

Coagulation factor V (FV) functions as both a procoagulant and anticoagulant factor in physiological processes. During coagulation, FVa acts as a cofactor to enhance the activation of prothrombin by coagulation factor X (FXa). In anticoagulation, FV serves as a cofactor for tissue factor pathway inhibitor (TFPIa) and activated protein C (APC) to exert anticoagulant activity [22–27]. There are three cleavage sites in FV for APC, namely Arg306, Arg506, and Arg679. APC catalyzes the cleavage of the Arg506 site, producing an intermediate with partial activity, and subsequently cleaves the Arg306 site to completely inactivate FVa. This results in the loss of FXa activity and inhibition of thrombin production. In addition, TFPIa and protein S (PS) also play a certain role in promoting decomposition [28–31]. The missense mutation at position 1691 of the FV gene (G to A substitution at base position 1691) leads to the substitution of Arg by Gln, known as the FV Leiden (FVL) mutation. Due to the Arg506 site is a favored cleavage site kinetically, FVa continues to express procoagulant activity and becomes resistant to APC cleavage, leading to APC resistance-related thrombotic events [32–34].

Different from conventional FV deficiency related to bleeding events, Castoldi et al. [35] reported recurrent venous thrombosis in a patient with FV deficiency. Whole exome sequencing found a novel FV missense mutation (Ala2086Asp, FV Besançon) resulting in an amino acid substitution in the FV C2 domain and reduced plasma levels. Although a small amount of FV is insufficient to exert the cofactor activity of APC and TFPI, it is still sufficient to activate prothrombin and maintain the coagulation reaction [36, 37]. In addition, similar effects have been observed in the FV Nara (Trp1920Arg) mutation [38]. This finding also highlights to clinicians that laboratory indicators alone may not fully reflect the clinical phenotype of patients with genetic mutations and therefore may not predict their expected outcomes accurately. The dosing regimen should not solely rely on laboratory indicators, but also consider the patient's clinical manifestations, imaging results, and other factors, to optimize their treatment and prevent potential complications. Overall, this highlights the importance of genetic screening for patients with suspected gene mutations.

Approximately 90% of FV mutations are FV Leiden (FVL). Mutations in FVL and prothrombin are the most common genetic factors associated with thrombophilia among individuals of Caucasian descent. The prevalence of heterozygous single-gene mutations is higher than that of homozygous and compound heterozygous individuals. In recent years, researchers have increasingly focused on the clinical distinctions between homozygous, heterozygous, and compound mutations, but consensus has not yet been reached. Botero et al. [39] found no significant difference in the incidence and recurrence rate of venous thromboembolism (VTE) between homozygous and heterozygous carriers of FVL. This may be attributed to the small number of homozygous carriers included in the study, introducing analysis bias. When individuals carry compound heterozygous mutations, there may be a synergistic effect, which increases the risk of thrombosis. In a study on tumor-associated thrombotic events, the incidence of homozygous or double heterozygous carriers of FV and FII gene mutations was significantly higher than that of single heterozygous mutations [40]. In a recent study on the double heterozygosity (DH) genotype for factor V Leiden and prothrombin G20210A, it was found that the DH genotype may occur as frequently as the FVL genotype and potentially increase the risk of developing VTE similarly [41]. In general, there are still several limitations in current research in this field. These confounding factors may affect the true outcomes, and the limited number of samples of homozygotes and compound heterozygotes introduces potential bias in data analysis [42]. Therefore, conducting a large-scale randomized controlled study with exclusion of clinical incentives is recommended as the optimal research approach. In clinical practice, it is also recommended to utilize activated protein C (APC) resistance testing in conjunction with DNA testing to identify FV Leiden and eliminate potential confounding factors that may obscure true results.

In recent years, other factors have gained increasing attention in the study of thrombotic diseases. The role of factor XI mutation is yet to be elucidated, and its clinical significance remains unknown. A gain-of-function mutation in the FIX gene at position 338, with arginine replacing leucine, was identified in a patient with thrombophilia in adolescence (FIXR338L, FIX Padua) [43]. Despite normal plasma levels of FIX, individuals with this mutation exhibit approximately 8 times higher activity levels than those with the wild type. Based on this finding, it was suggested that the stability of FIXaR338L binding to PS decreased, and the ability of PS to inhibit FIXaR338L decreased, suggesting an increased risk of thrombosis [44]. In vitro studies have shown that supplementation of exogenous PS improves the patient's condition, providing new ideas for the treatment of FIX-related thrombophilia. The 1813 site in the A3 subunit of FVIII is responsible for binding to FIXa and dissociation of the A2 subunit from FIXa. The K1813A mutation enhances the activity and structural stability of the FVIII cofactor, resulting in significantly increased coagulation potential [45]. Although this mutation is considered to be useful for protein and gene therapy in patients with hemophilia A, its role in contributing to thrombotic events remains to be studied. Individuals carrying the β-fibrinogen 455A allele exhibit elevated plasma fibrinogen levels and an increased risk of arterial thrombosis, which is more common in the Asian population [46, 47]. This allele significantly associates with susceptibility to cardiogenic stroke, ischemic stroke, and coronary heart disease.

### Coagulation inhibitor abnormalities

Antithrombin (AT) is a glycoprotein synthesized by hepatocytes and vascular endothelial cells, functioning as a major component of the natural anticoagulant system. Under normal circumstances, AT has minimal direct anticoagulant effect due to the absence of heparin in plasma [48]. However, during the process of anticoagulation, heparin binds to the heparin-binding region at the N-terminus of AT, inducing a conformational change and exposing the reactive central loop (RCL). This results in an approximately 2000-fold increase in the anticoagulant activity of AT. The C-terminal protease inhibitory active region of AT then covalently binds to the serine residue of the thrombin active center, forming a stable heparin-AT-thrombin complex. This complex weakens or completely inhibits the activity of thrombin. Subsequently, heparin dissociates from the complex, allowing it to recombine with another molecule of AT for repeated use. In addition to its effects on thrombin, AT also inhibits other coagulation factors such as FXIa, Xa, XIa, and XIIa, all of which are serine proteases. Furthermore, AT promotes the dissociation of the coagulation factor FVIIa-tissue factor complex, inhibiting the initiation of the exogenous coagulation pathway.

The incidence of antithrombin deficiency ranges from about 0.02% to 0.2%, increasing the susceptibility to thrombosis. The first report of a family with congenital antithrombin deficiency thrombophilia was by Egeberg in 1965. Since then, studies have been conducted on families with hereditary antithrombin deficiency. Over 300 mutations have been identified, with 80% of antithrombin defects caused by mutations in the SERPINC1 gene. Mutation types include point mutations, splicing, and small fragment insertion/deletion, with over 90% being point mutations. Missense/nonsense mutations account for 55.0% of all mutations [49].

**Fig. 3:**
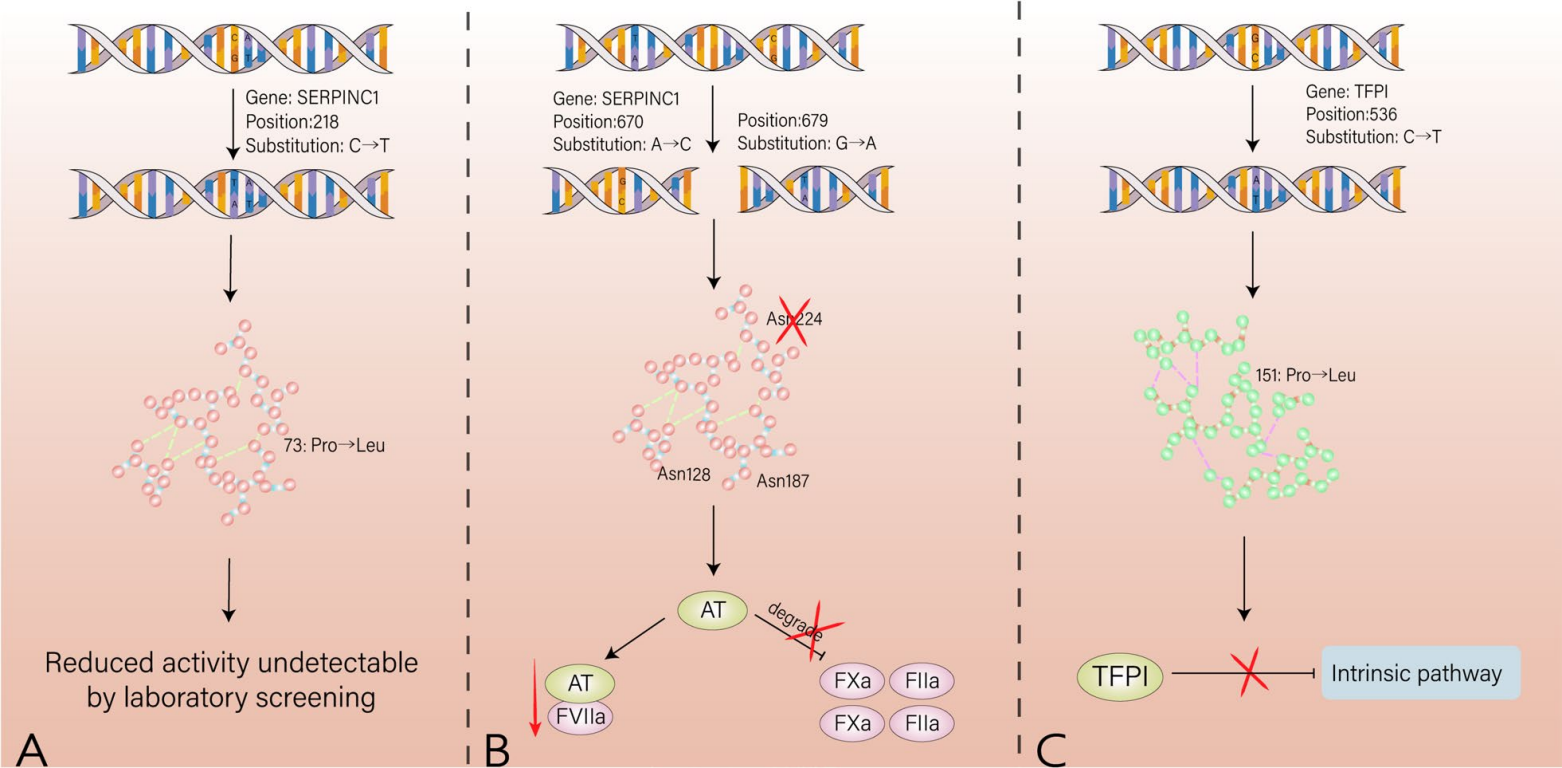
Thrombosis caused by abnormal coagulation inhibitors. **A** 80% of the antithrombin deficiency is caused by SERPINC1 gene mutation. **B** In addition, *N*-glycosylation defects can lead to a decrease in its anti-FXa and anti-FIIa activity. **C** Tissue factor pathway inhibitor (TFPI) can specifically inhibit the tissue factor pathway and hinder the initiation of coagulation reaction. Single nucleotide substitution (C536T) in exon 7 will lead to a relative increase in the risk of venous thrombosis

AT defects are typically categorized into two types based on activity and plasma antigen levels. Type I, known as the classical defect type, is characterized by a disorder in AT synthesis, resulting in a parallel decrease in activity and plasma antigen levels. Type II is merely characterized by reduced AT activity (shown in Fig. 3). Within Type II defects, there are three subtypes based on different mutation sites: reaction site defect type II (II/RS), heparin binding site defect type II (II/HBS), and pleiotropic defect type II (II/PE). The specific types of SERPINC1 mutations and the different subtypes of AT deficiency can influence the clinical phenotype of hereditary AT deficiency to some extent, particularly in terms of varying risks and locations of thrombosis. For example, SERPINC1 missense mutations are associated with a lower risk of venous thromboembolism, and nearly all type II AT deficiencies are caused by missense mutations. Although the risk of venous thromboembolism is lower in type II HBS AT deficiency compared to other types, these patients have a higher risk of arterial thromboembolism [50].

Transient antithrombin (AT) deficiency is a newly defined concept, referring to the clinical effects caused by mutant genes at specific time points due to external factors [51]. Patients with this type of deficiency have a higher incidence of arterial thrombosis events. AT, being a glycoprotein, possesses four N-glycosylation sites (Asn128, Asn167, Asn187, Asn224). The majority of plasma AT is fully glycosylated (α-AT, 90%), while a small portion exhibits a deficiency in the Asn167 glycosylation side chain (β-AT, 10%). Due to its high affinity for heparin, β-AT becomes the primary coagulation inhibitor in the body in a low-abundance form [52, 53]. *N*-glycosylation defects can also cause antithrombin deficiency, suggesting the existence of a new mechanism of thrombophilia. When a missense mutation is located close to or within the *N*-glycosylation sequence, it impairs the glycosylation of Asn224 and leads to decreased anti-FXa and anti-FIIa activity of β-AT. Since β-AT constitutes only a small proportion in plasma, conventional laboratory tests primarily screen the ability of AT to inhibit exogenous FIIa or FXa, resulting in the under-recognition of this defect. However, the level of FVIIa-AT complex decreased, the exogenous coagulation pathway was significantly enhanced, and the plasma showed a hypercoagulable state (shown in Fig. 3). Furthermore, glycosylation defects alter the folding form and stability of AT, making it more susceptible to external conditions such as temperature and alcohol. This clinical manifestation can be observed as transient antithrombin deficiency[51]. The incidence and severity of thrombosis in these patients are lower than those with continuous deficiency, but arterial thrombosis events are relatively common.

The tissue factor pathway inhibitor (TFPI) plays a crucial role in inhibiting the tissue factor pathway and hindering the initiation of coagulation reactions. A single nucleotide substitution (C536T) on exon 7 results in the substitution of proline by leucine (Pro151Leu) at amino acid 151 of the protein, which increases the risk of venous thrombosis (shown in Fig. 3). Although the biochemical regulation of TFPI is obvious, the function or concentration determination of its plasma level cannot accurately reflect the defects of TFPI [54]. There is also no evidence indicating clinical relevance of such mutations. Therefore, the clinical significance of TFPI defects remains to be elucidated.

### Abnormal inactivation of fibrinolytic system

**Fig. 4:**
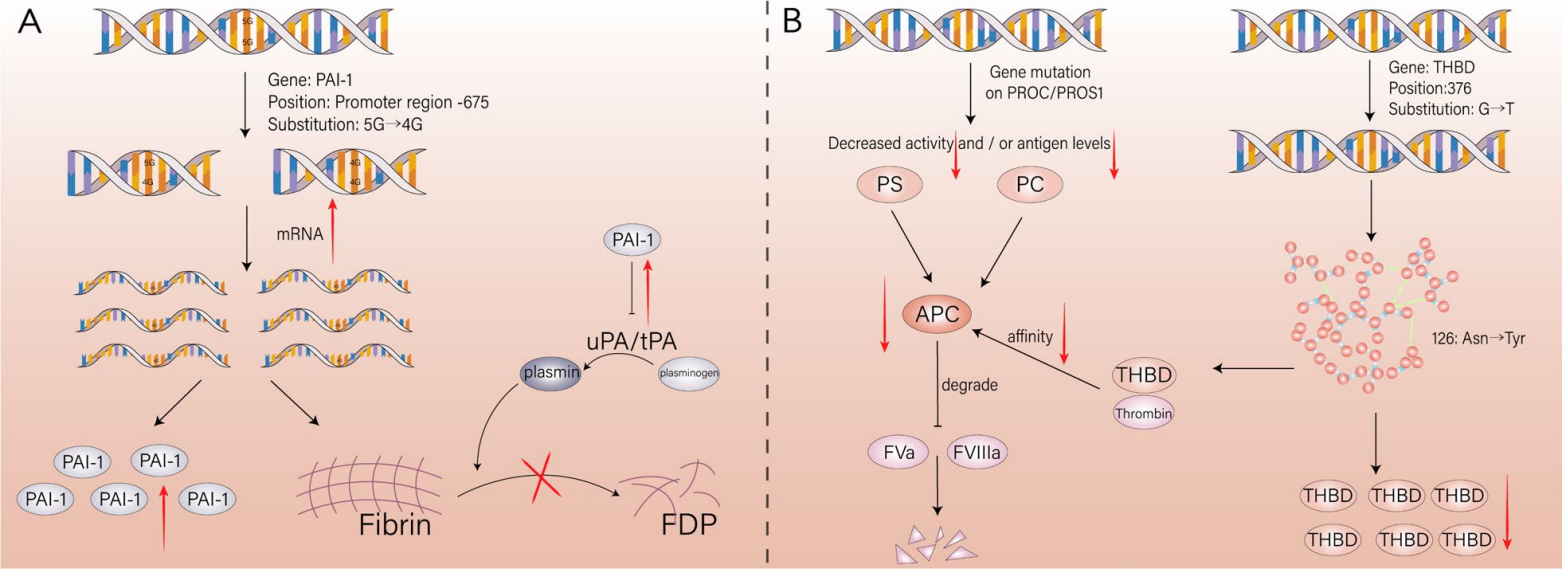
Thrombosis caused by abnormalities of fibrinolytic system and protein C system. **A** The carrying of 4G gene in the promoter region of PAI-1 coding gene increased the transcription level, protein expression and activity of PAI-1, and inhibited the dissolution of fibrin clot. **B** Mutations in protein C and protein S coding genes cause decreased activity and/or decreased antigen levels. Mutations in thrombomodulin will lead to a decrease in the affinity of the thrombin complex to protein C and a delay in the co-activation effect

Plasminogen activator inhibitor-1 (PAI-1) serves as the primary inhibitor of endogenous plasminogen activators, namely tissue plasminogen activator (tPA) and urokinase (uPA), effectively impeding the dissolution of fibrin clots. Under physiological conditions, PAI-1 assumes responsibility for regulating fibrinolytic activity and maintaining a delicate balance between fibrinolysis and coagulation within the body. Thorough investigations into the coding genes of PAI-1 have predominantly focused on the -675 4G/5G polymorphism found in the gene's promoter region. Notably, the presence of the 4G allele significantly elevates transcription levels, protein expression, and PAI-1 activity (shown in Fig. 4). While some studies argue against any correlation between the 4G allele and coronary heart disease or myocardial infarction, with no observed prothrombotic effects in FVL carriers [55, 56], Balta et al. [57] have reported a distinct association linking 4G allele carriers to visceral vascular thrombosis, particularly portal vein thrombosis. Furthermore, continuous monitoring of PAI-1 activity and antigen levels in patients undergoing anticoagulant therapy after experiencing deep vein thrombosis (DVT) revealed a substantial increase in PAI-1 activity among carriers of the 4G allele, accompanied by a relative surge in thrombosis frequency [58]. A comprehensive meta-analysis has pinpointed the polymorphism within the PAI-1 promoter region as a susceptibility locus for thrombosis [59], potentially influenced by the regulation of lipid metabolism or interactions with other genetic mutations involved in thrombosis. Inconsistencies or even contradictory outcomes across different studies may be attributed to variations in population, limited sample sizes, or divergent susceptibilities under different disease states. Due to the heterogeneity of these findings, the use of PAI-1 detection in clinical practice is not yet viable. Further research is needed to clarify its potential for independent cardiovascular event prediction and accurate prognosis forecasting. Recent discoveries have shed light on the involvement of polymorphisms within the 3'-UTR region of the PAI-1 gene, further enhancing our comprehension of PAI-1 gene polymorphisms and their implications in stroke risk regulation [60].

### Protein C system abnormalities

The protein C system mainly includes protein C (PC), protein S (PS), thrombomodulin (THBD), endothelial protein C receptor (EPCR), activated protein C receptor (APCR), and protein C inhibitor (PCI). Synthesized in the liver, protein C initially exists in the plasma as an inactive zymogen. Thrombin binds to THBD on the vascular endothelial cell membrane to form a thrombin-THBD complex. The procoagulant activity of thrombin is masked and neutralized. Thrombin degrades PC to form activated protein C (APC), and EPCR and APCR can enhance the activation [61]. APC inactivates FVIIIa and FVa with the help of the non-enzymatic cofactor PS, limiting the coagulation reaction [62]. APC was cleared by PCI, α1-antitrypsin (α1-AT), α2-macroglobulin (α2-MG) and α2-antiplasmin (α2-AP). At present, there is no clear conclusion on the specific mechanism of how PS enhances PC, but it is assumed that PS binds to the Gla and EGF1 domains of APC on the surface of a high-affinity phospholipid membrane to stabilize its active conformation. In addition, PS can enhance the inhibition of FXa activity by TFPI, and even PS containing zinc ions can directly bind to tissue factor and inhibit the production of FXa [63].

Although rare, genetic defects in PC and PS serve as independent risk factors for VTE along with FV Leiden, suggesting a predisposition to severe thrombophilia (shown in Fig. 4). These genetic defects in the protein C (PROC) and protein S (PROS1) encoding genes are polymorphic, encompassing gene polymorphisms, plasma phenotypic variations, and clinical phenotypic variations. Studies have identified heterozygous mutations in the protein C system, while homozygous mutations are rare and typically associated with neonatal fulminant purpura, a life-threatening condition. Hotspot mutations such as Glu67Ala, Arg561Trp, and Tyr560* have been observed in the Chinese population, whereas Lys196Glu is common in the Japanese population [64, 65]. Therefore, there are variations in genetic factors related to coagulation not only between the Caucasian and East Asian populations, but also within the same population.

As a protease, protein C (PC) is composed of four main domains: Gla, EGF1, EGF2, the activating peptide and the enzymatic serine protease domain. Recent studies have revealed a series of mutations in genes encoding PC, which disrupt its stable structure and subsequently reduce its anticoagulant activity. PC deficiency can be categorized into two types. Type I deficiency is characterized by mutations in the Gla or serine protease domain, leading to decreased levels of both antigen and activity, such as p. Ala178Pro and p.Pro275Ser. Type II deficiency is characterized by reduced activity levels only, such as p. Arg189Trp and P.Lys193del. Type II deficiency can be further subdivided into type IIa, caused by mutations at the APC enzyme cleavage site, and type IIb, caused by mutations at other functional sites that affect the interaction between PC and other molecules. Similarly, different mutations in protein S (PS) result in distinct plasma phenotypes. Quantitative defects in PS can occur throughout the entire PROS1 region, while qualitative defects are typically limited to the Gla and EGF-like domains. It is hypothesized that the active center of PS is primarily located within the Gla and EGF domains.

According to statistical data, around half of individuals carrying protein S (PS) mutations experience clinical symptoms, with the majority of these symptoms manifesting before the age of 55. Notably, patients with mutations in the SHBG-like domain tend to develop thrombosis at an earlier age [66]. Zhang et al. [67] found a frameshift mutation (c.74dupA) of PROS1, and the proband exhibited hypoxia induction in comparison to asymptomatic carriers within the same family. Based on these findings, it is speculated that this mutation does not display a procoagulant tendency when present in isolation, and its pathogenicity may be influenced by the presence of other inducing factors.

Thrombomodulin (thrombomodulin, THBD) comprises an N-terminal C-type lectin-like domain, six epidermal growth factor (EGF)-like modules, and a serine/threonine-rich region. During anticoagulation, the EGF-like structure binds to thrombin and alters its substrate specificity, transitioning from a procoagulant to an anticoagulant state. In addition to functioning as a cofactor for protein C, thrombomodulin can activate thrombin to promote the activation of fibrinolysis inhibitors and degrade inflammation-related high mobility group box 1 (HMGB1), thereby participating in fibrinolysis and anti-inflammatory processes. Previous studies have primarily focused on mutations within the EGF-like and serine/threonine-rich domains of thrombomodulin. More recently, several studies have demonstrated a close relationship between single nucleotide polymorphisms in the THBD gene and thrombotic diseases [68–72]. Furthermore, heterozygous mutations in the C-type lectin domain (c.376G > T) result in the substitution of aspartic acid with tyrosine at residue 126, altering the molecular conformation of thrombomodulin and leading to decreased plasma antigen levels accompanied by functional defects. This mutation reduces the affinity between the thrombin-thrombomodulin complex and protein C while also delaying the co-activation effect of protein C, thereby increasing susceptibility to thrombosis (shown in Fig. 4). Clinically, patients with this mutation experience recurrent arterial and venous thrombosis [73].

In recent years, there has been growing interest in understanding the relationship between mutations in other molecules within the protein C system and thrombotic diseases. One notable example is the homozygous mutation of α1-antitrypsin Z (Glu342Lys), which has shown a 2.2-fold correlation with venous thromboembolism (VTE) [74–76]. This correlation is comparable to that of the prothrombin G20210A mutation. However, the specific mechanism underlying this correlation remains to be elucidated [76]. In summary, while protein C serves as a core molecule, various coagulation-related substances indirectly contribute to promoting thrombosis by affecting the function of protein C. Therefore, further exploration of protein C's role in the blood system is necessary.

### Other factors

Abnormalities in the renin–angiotensin–aldosterone system, which regulates vascular contraction and intimal hyperplasia, may also lead to thromboembolism. The renin gene on chromosome 17 has an insertion (I)/deletion (D) polymorphism, resulting in three genotypes: II, ID, and DD. Many studies have observed that the DD genotype is associated with a high risk of thrombosis. In patients undergoing dialysis, carriers of the DD genotype have increased levels of PAI-1, resulting in a state of reduced fibrinolysis and a significantly increased risk of thrombosis [77]. However, the relationship between these factors has not been fully elucidated, and a prospective, multicenter study with a large sample size is needed to establish a definitive relationship. Additionally, differences in the incidence of VTE have also been observed based on different blood types among individuals, with a significant increase in the risk of VTE when FVL coexists with non-O blood types [78].

Pulmonary embolism (PE) patient with thrombophilia have a tendency to develop deep vein thrombosis, particularly in patients with deficiencies in PC and PS [79]. Therefore, it is highly significant for the clinical prognosis to identify such inherited disorders and provide appropriate preventive measures. Given the high cost of genetic testing, it is not recommended for all PE patients to undergo genetic screening. The focus should be on individuals with a family history or those who are not predisposed to PE. In a prospective family cohort study [80], individuals with inherited deficiencies of natural anticoagulants (AT, PC, PS) had a nearly five times higher risk of arterial thrombosis compared to those without these deficiencies, with young individuals exhibiting a higher susceptibility. Therefore, early, timely, and effective identification of genetic factors associated with thrombotic disorders can help prevent thrombosis, improve patient prognosis, and avoid adverse events. This requires the development of high-throughput, high-sensitivity detection technologies.

## Clinical commonly used risk prediction scale

While not all mutant carriers experience thrombotic events, the presence of acquired risk factors and environmental factors can significantly increase their probability. Common risk factors for venous thromboembolism (VTE) include male gender, diabetes, obesity, smoking, mutations, use of oral contraceptives and hormone replacement therapy, long-distance flights, severe acute respiratory distress syndrome, coronavirus type 2 infection, trauma and fractures, pregnancy, immobilization, antiphospholipid syndrome, surgery, and cancer. Currently, anticoagulant therapy is not recommended for mutation carriers. However, for patients with risk factors, it is crucial to have an effective risk assessment system in place for predicting and stratifying risks. This would enable doctors to identify high-risk patients promptly and initiate timely anticoagulant therapy, while also avoiding unnecessary anticoagulation in low-risk patients to prevent bleeding events. We reviewed the risk assessment models (RAMs) commonly used in clinical practice, and summarized the application conditions, advantages and limitations. These models are only applicable to hospitalized surgical and medical patients or ambulatory patients with cancer prior to chemotherapy (shown in Table 1). On this basis we outline the qualities that an ideal assessment model should have.

**Table 1:** Commonly used thrombus risk prediction models

Model	Participant	Application	Validation method	Shortcoming	Research type	Result	Refs.
Caprini Score (1991)	Patients undergoing general, urological, orthopaedic, gynaecological and head and neck surgery	Determine appropriate thromboprophylaxis based on risk stratification	No validation done	Failure to perform effective validation	Prospective study (n = 538)	37.2% of the patients received prophylactic regimens (low-risk cases: 10%; moderate-risk cases: 42.1%; high-risk cases: 76%)	[81]
Padua Score	Consecutive internal medicine patients meeting inclusion criteria	Distinguish between admitted medical patients at high and low risk of venous thromboembolic complications and implement adequate thromboprophylaxis in high-risk patients	Independent, blinded assessment of outcome, but not validated	Lack of external validation. Subsequent validation by others' studies showed greater variability	Prospective cohort study (n = 1180)	To assess the adjusted risk ratio for VTE in high-risk patients with adequate in-hospital thromboprophylaxis compared with high-risk patients without (VTE HR, 0.13; 95% CI 0.04–0.40); and the adjusted risk ratio for VTE in the latter compared with low-risk patients (VTEHR 32.0; 95% CI 4.1–251.0)	[82]
Khorana Score	Outpatients with solid tumors receiving chemotherapy	Selection of outpatient cancer patients at high risk of venous thromboembolism	Split sample method to obtain an independent validation cohort without external validation	Heterogeneity exists according to the type of species, showing instability in the clinical application of lung cancer, with a higher residual risk in the intermediate- and low-risk groups of patients	Multi-centre prospective observational study	Outpatients with solid tumors receiving chemotherapy	[83]

### Surgical inpatients

The Caprini risk assessment scale has long been used for evaluating the risk of venous thromboembolism (VTE) in surgical patients. Originally developed in 1991, the scale underwent revisions in 2005 and 2010 to create a more personalized risk assessment approach [81]. Consisting of approximately 37 risk factors, each assigned a weight of 1–5 points, the scale categorizes patients into four risk groups based on their total score. Correspondingly, recommended therapies are provided for each group. Periodic reassessments are conducted at specific intervals. In an orthopedic patient cohort, a prospective study evaluated the predictive ability of the modified 2005 and 2010 versions of the Caprini scale for deep vein thrombosis (DVT) upon admission. The study found that the 2005 version demonstrated higher sensitivity compared to the 2010 version, while specificity remained consistent [84]. However, it is important to note that this study did not objectively verify the presence of DVT using imaging techniques (such as Doppler ultrasonography) for all patients. Additionally, it did not evaluate the dynamic changes in risk factors from the time of surgery to the time of discharge, potentially overlooking the influence of new factors that may arise during this period.

Lobastov et al. [85] conducted a meta-analysis to investigate the relationship between the Caprini risk score threshold and the occurrence of venous thromboembolism (VTE). The results revealed that a score of 7–11 points had significant predictive value for VTE occurrence. However, similar positive results were not observed in obstetrics, bariatric surgery, neurosurgery, and transplant patient populations. The lack of consistent results may be attributed to the unique characteristics of these diseases and the importance of effective patient management strategies. In another prospective cohort study, the reliability and effectiveness of a modified Caprini score were evaluated. This revised version included an ultra-high-risk group, defined by a score exceeding 8 points. Patients with scores higher than 8 points were found to have a significantly increased risk of developing VTE, which was consistent with the findings reported by Pannucci and Zhou in their respective studies [86–88]. These results highlight the need for detailed stratification of high-risk patients to more accurately assess their level of risk. Interestingly, these findings differ from those reported in Western populations by Caprini. It is assumed that the type of population plays a crucial role in the occurrence of VTE. However, this study solely relied on clinical manifestations as scoring criteria, omitting important indicators such as FVL and serum homocysteine levels. Additionally, routine screening for asymptomatic VTE patients was not performed, which could potentially lead to underreporting of incidence rates. These factors may contribute to the inconsistencies observed in the results.

To establish a more comprehensive understanding of the relationship between the Caprini risk score and VTE occurrence, future studies should consider incorporating a broader range of indicators and implementing routine screening measures. By addressing these limitations, we can gain deeper insights into the risk assessment of VTE in different patient populations.

### Internal medicine inpatients

The Padua rating scale is frequently used for medical inpatients. Barbar et al. proposed the scale in 2010 and validated it in a prospective cohort study. The scale consists of 20 points, with scores > 4 classified as high-risk patients for VTE, while scores < 4 were classified as low-risk patients [82]. Since its release, many studies have verified its reliability. However, variations in subject composition and study design could be attributed to the large differences reported among these studies. Retrospective case–control studies often lead to a relatively high prevalence in the subjects, resulting in increased sensitivity and an overestimation of the scale's predictive performance. Additionally, disparities in sample sizes, researchers' subjective factors, ethnic disparities, and regional differences may also cause variations. Moumneh [89] externally validated the predictive ability of Caprini, Padua, and IMPROVE (Internal Medicine Patient Bleeding Risk Assessment Scale) scores for 3-month VTE in medical inpatients. The results showed that there was no significant difference in the area under the receiver operating characteristic curve (AUC) of the three, which was about 0.60–0.65. None of them showed a better ability to identify risk than the single predictor of age. This inevitably raises doubts about the necessity of clinical application of the risk assessment scale. We speculate that these risk prediction scales may exhibit superiority in specific disease subgroups, and this sensitivity may be masked in the evaluation of overall medical patients. Meanwhile, due to the retrospective collection of data, the BMI of many patients is artificially supplemented because it is not available, which may deviate from the true value. Other studies have shown that the predictive ability of the IMPROVE assessment model improved when the combined elevated D-dimer was analyzed together [90], suggesting that scientific inclusion of risk factors can help predict disease risk better.

### Patients with cancer

Tumor in situ compression leads to blood stasis, and the secretion of cytokines leads to hypercoagulable state of blood, which makes tumor patients one of the high-risk groups of thrombosis. As the first fatal complication of cancer, VTE leads to about 9% of total deaths. Since the variables of Khorana score (KRS) are clinically accessible and routinely collected data, the current guidelines recommend the use of KRS to assess the risk of VTE formation for new cancer outpatients receiving chemotherapy [83]. However, the lack of an ideal patient data set for external validation has limited the scale development process, which solely relied on the split-sample method to construct and validate the model. Numerous studies have demonstrated the effectiveness of KRS. However, due to variations among studies and the subjective interpretation by different doctors, the accuracy of KRS remains unclear. KRS is designed for a heterogeneous population with various cancer types, and its evaluation efficacy differs across specific cancer types. Furthermore, variables occurring during the treatment process, such as chemotherapy drug types, adverse reactions, and vascular invasive procedures, may also influence the occurrence of VTE. Therefore, additional relevant factors should be incorporated to enable KRS to provide a dynamic evaluation throughout the entire chemotherapy process.

The role of prethrombotic genetic factors in the occurrence of venous thromboembolism (VTE) in cancer patients has garnered increasing attention in recent years. In a study of cancer outpatients receiving chemotherapy, researchers found that FVL mutations and non-O blood types were significantly associated with VTE, and that prophylactic anticoagulant therapy effectively reduced the risk of thrombotic events in such populations [91]. Guman et al. [92] used a multi-gene VTE risk score to assess the risk of cancer-related VTE, and compared it with KRS. The results showed that the score based on multiple genes showed more stable performance in thrombosis prediction. After the tumor type is included in the evaluation factors, the performance is better. Therefore, it is of clinical significance to determine whether the patient has a coagulation-related genetic mutation or whether it is a non-O blood group before chemotherapy.

Based on the above, Muñoz et al. [93] developed a new VTE scoring model (ONCOTHROMB score) for cancer outpatients, which combined genetic factors and clinical factors. External validation of this score in 263 patients showed promising results, but further studies are needed to confirm its applicability and accuracy.

### Other thrombotic risk assessment models

Besides the three commonly used thrombotic risk assessment models mentioned above, there are also specific scales in clinical practice for assessing thrombotic risk under certain disease states. Pulmonary embolism (PE) can present with varied symptoms ranging from asymptomatic to severe symptoms such as dyspnea, chest pain, and hemoptysis, leading to high rates of misdiagnosis and underdiagnosis due to their nonspecific nature. Given the high mortality and disability rates associated with PE, prevention for this condition is crucial. The simplified Wells score and the revised Geneva score are frequently used to predict the likelihood of PE [94, 95]. Both scoring systems contain similar items; however, the Wells score includes the subjective factor of "alternative diagnosis less likely than PE," which carries a high subjective component and scoring, potentially leading to errors in clinical use. In contrast, the revised Geneva score eliminates arterial blood gas analysis and chest X-ray items from the original scoring system and introduces scoring items such as malignancy, hemoptysis, unilateral lower limb pain and swelling, and lower limb deep vein tenderness, replacing subjective factors with objective measurements and thereby enhancing objectivity. Additionally, this scoring system has undergone extensive external validation and serves as an effective tool for assessing PE risk in internal medicine inpatients. Furthermore, the Pulmonary Embolism Severity Index (PESI) is used to assess the severity of PE [96]. Risk stratification for thromboembolism is crucial as it directly impacts treatment strategies and prognosis. By assessing clinical features and risk factors, high-risk and low-risk patients can be identified. High-risk patients may require urgent treatment to reduce mortality risk. Risk stratification also optimizes resource utilization, ensuring timely treatment and monitoring to decrease complications such as recurrent embolism. Effective risk stratification improves treatment outcomes and facilitates personalized care implementation.

For patients with thrombosis, assessing the risk of bleeding is crucial when deciding on anticoagulant therapy. Anticoagulants prevent blood clotting to prevent thrombus formation or reduce thrombus size. However, this also decreases the blood's clotting ability, increasing the risk of bleeding. Considering the bleeding risk helps doctors choose treatments more cautiously to prevent thrombosis while minimizing bleeding risks. It's worth noting that there are currently no effective predictive models to accurately assess bleeding risk in anticoagulated patients.

## The application of AI in thrombotic diseases

**Fig. 5:**
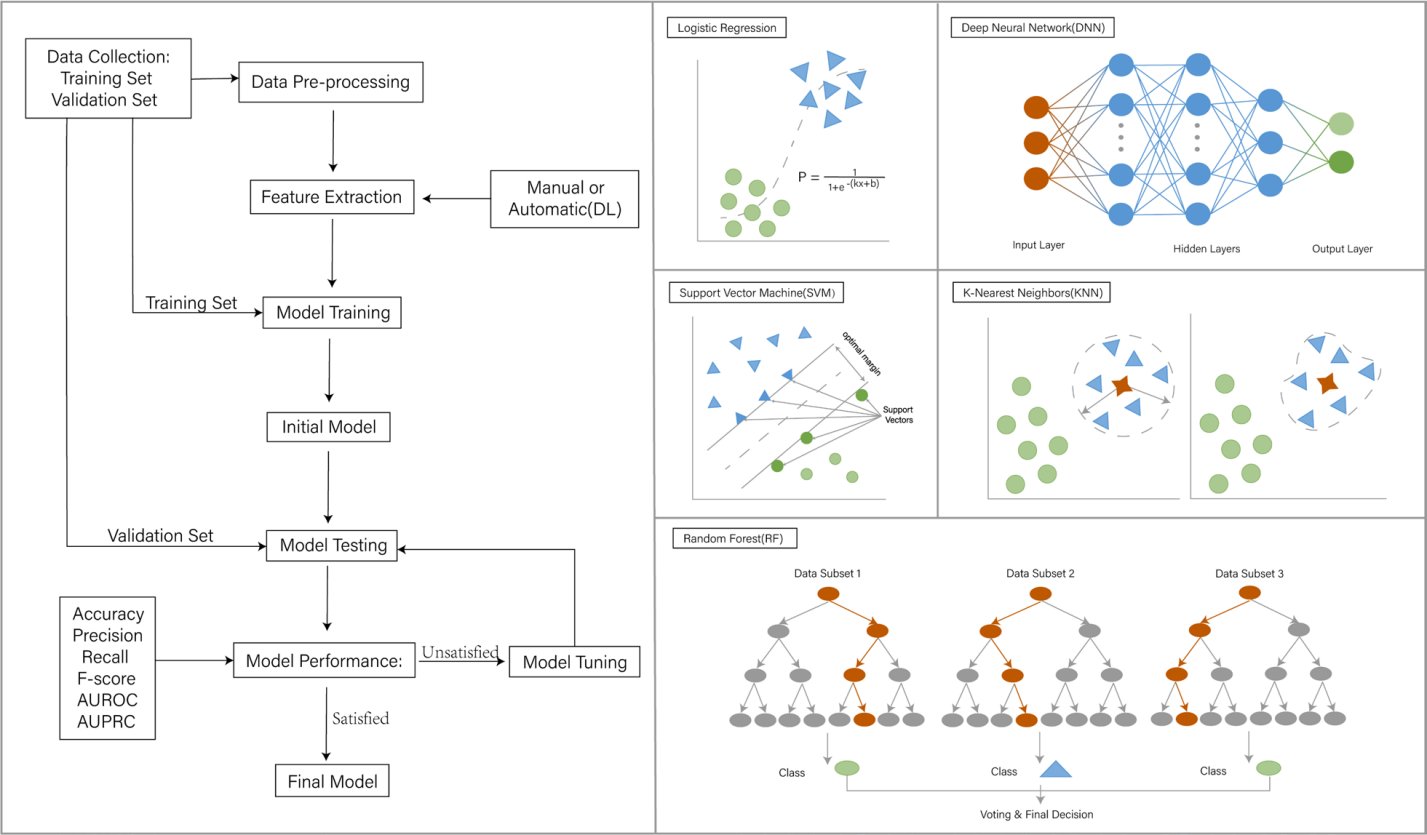
The basic process of using artificial intelligence to build medical models, and some common algorithms. The preprocessed data is usually divided into three parts: training set, validation set and test set. Researchers choose specific algorithms according to different application scenarios. After using the training set to learn and master the relevant knowledge, the corresponding models can be obtained. The validation set is used for verification, and the best model is selected after quantifying the score

Since its inception in 1956, Artificial Intelligence (AI) has undergone continuous development. With the expansion of research in various disciplines, AI has become an interdisciplinary frontier discipline, with increasingly enriched application methods and implementation approaches. Leveraging big data and powerful hardware/computing capabilities, machine learning (ML) enables the establishment of a universal and consistent research model. AI can be applied to natural language processing, computer vision, robotics, intelligent search, data mining and expert system. Machine learning is the core branch and main implementation method of AI, including supervised learning, semi-supervised learning, unsupervised learning and reinforcement learning. Deep learning represents a further advancement in machine learning, utilizing multi-layer neural networks to mimic the neural mechanisms of the human brain for data analysis and interpretation. Deep learning enables the analysis of larger and more complex datasets, albeit with higher demands on computer computing power, making it the most concerned learning method currently. Machine learning comprises three key components: models, strategies, and algorithms. Researchers select specific learning methods based on different application scenarios and utilize large-scale datasets as training sets to acquire and master relevant knowledge, thereby constructing appropriate models. The selection of the best model is determined through validation using an independent validation set. Prior to actual deployment, the model is compared with existing models/systems to demonstrate its comparative advantages. Overall, the continuous development and advancement of AI, particularly within the field of machine learning, has paved the way for its practical applications (shown in Fig. 5).

**Fig. 6:**
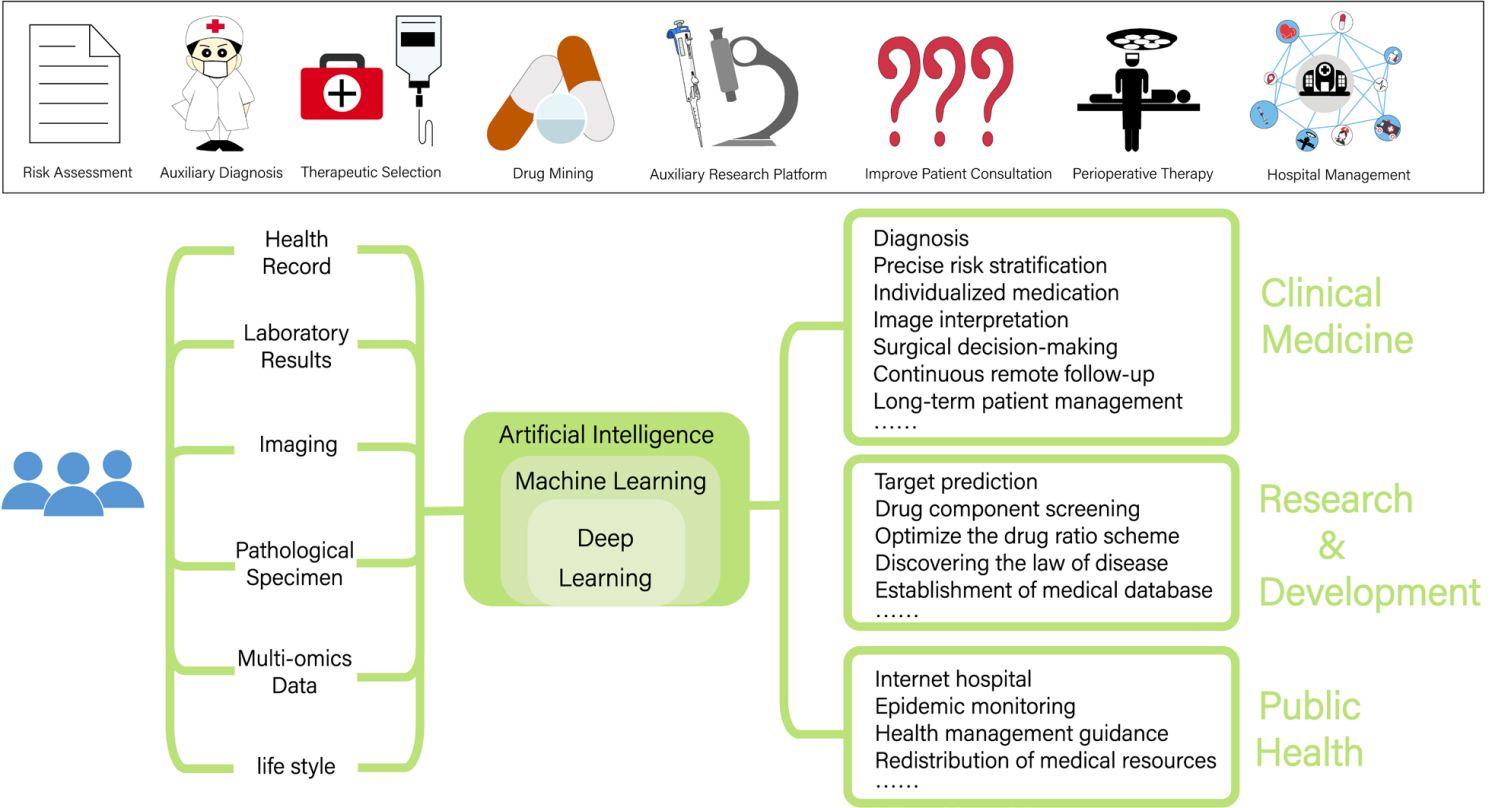
Application of AI in the medical field. The application fields of medical artificial intelligence include but are not limited to improving clinical diagnosis and treatment, assisting medical research, and promoting public health management. Application of clinical diagnosis and treatment: risk assessment, auxiliary diagnosis, clinical management, improvement of patient consultation, determination of surgical indications, intraoperative decision-making, postoperative management, etc. Auxiliary medical research: disease target prediction, drug component screening, discovery of disease rules, etc. Public health management: Internet hospitals, epidemic monitoring, optimization of medical resource allocation

In recent years, the potential benefits of AI in clinical practice have been gradually explored, including rapid processing of large-scale data, correlation analysis to remove redundant variables and avoid model over-fitting, and the use of consistent, homogeneous, and objective evaluation criteria to enhance diagnostic accuracy and specificity. The construction process of a clinical model primarily involves screening important covariates, filtering the input sample data, constructing the model using machine learning techniques, and validating its internal and external validity. When conditions permit, continuous follow-up and dynamic adjustments to the model are preferable. Medical artificial intelligence finds application in various fields, including but not limited to risk assessment, auxiliary diagnosis, clinical administration, improving patient consultations, determining surgical indications, making intraoperative decisions, and postoperative management (shown in Fig. 6).

### Risk identification

Machine learning combined with clinical data can assist in identifying disease-related risk. Through rigorous data training, machine learning models can identify complex interactions between variables and discover predictive variables with clinical significance. By comparing with existing scoring systems and conducting thorough external validation, the generalizability of the machine learning model can be thoroughly demonstrated. Pituitary adenomas exhibit significant heterogeneity, making it challenging for traditional scoring systems to accurately predict postoperative outcomes. Hollon et al. [97] used four supervised machine learning algorithms, including naive Bayes, logistic regression, support vector machines, and random forest, to construct and cross-validate models. The test results demonstrated an accuracy of up to 87%. Furthermore, exploratory data analysis revealed risk factors for common postoperative complications, such as youth and obesity.

Thrombotic disease is a leading cause of global mortality, with risk factors encompassing both genetic and acquired factors. Early identification of individuals at high risk of thrombosis and the provision of appropriate preventive and therapeutic interventions are crucial. Machine learning serves as a powerful tool for thrombosis risk assessment, accurately identifying individuals who require personalized anticoagulation therapy. Previous studies have developed more accurate prediction models for venous thrombosis using machine learning methods [98] even without prior knowledge of relevant risk factors, highlighting the advantage of machine learning in uncovering potential risk factors. Subsequent generation of a thrombotic risk predictor using support vector machines and stochastic optimization in cancer outpatients showed more confidence than the KRS rating scale [99]. The significance of optimizing the clinical attribute group in the selection of predictors was also highlighted. Liu et al. [100] further developed a risk model for peripherally inserted central catheters in cancer inpatients, incorporating the PAI-1 genotype as a variable. To mitigate overfitting tendencies and ensure the model's robustness, a random decision forest was utilized. It is widely acknowledged that older age is an independent risk factor for thrombosis. Studies have revealed that the composition of risk factors for venous thromboembolism (VTE) differs between young and middle-aged inpatients and the elderly. Building on this, Liu [101] employed five algorithms to analyze clinical data from both patients and healthy individuals, effectively identifying the primary predictor of adverse outcomes in this population. Accurate and efficient prediction of mortality rates among patients in intensive care units (ICUs) is of great clinical significance. Such predictions aid in determining appropriate anticoagulation regimens for doctors and facilitate the rational allocation of ICU healthcare resources in the short term. Furthermore, they contribute to improving patient quality of life and reducing medical expenses. For ICU patients with VTE or cancer, two machine learning models—automated and customized—were developed to predict early and late mortality, respectively [102]. At the same time, a new biomarker was discovered.

The application of artificial intelligence in medicine covers various aspects from the entire disease process to prognosis assessment and recurrent risk prediction, providing doctors with more effective management measures to improve clinical outcomes. To overcome the limitations of existing statistical models, Martins et al. utilized artificial neural networks (ANNs) to predict recurrent venous thromboembolism, demonstrating through fivefold cross-validation that ANNs have higher accuracy and applicability compared to traditional scoring models. On the other hand, Mora et al. employed multiple machine learning methods including neural networks and logistic regression to study the prognosis of acute pulmonary embolism patients who prematurely stopped anticoagulant therapy. The results showed that neural networks (ML-NN) outperformed traditional logistic regression in predicting composite outcomes for patients. Therefore, the application of artificial intelligence technologies such as neural networks in medicine not only enhances the accuracy of prognosis assessment but also opens new possibilities for personalized healthcare, which is crucial for improving clinical outcomes for patients. Compared to other artificial intelligence learning methods, neural networks have the capability to model non-linear relationships. Therefore, their ability to extract information from complex data is stronger and more flexible. In addition, neural networks also excel in automatic feature learning, multi-task learning, and other advantages, which make them more efficient in practical applications.

### Auxiliary diagnosis

**Fig. 7:**
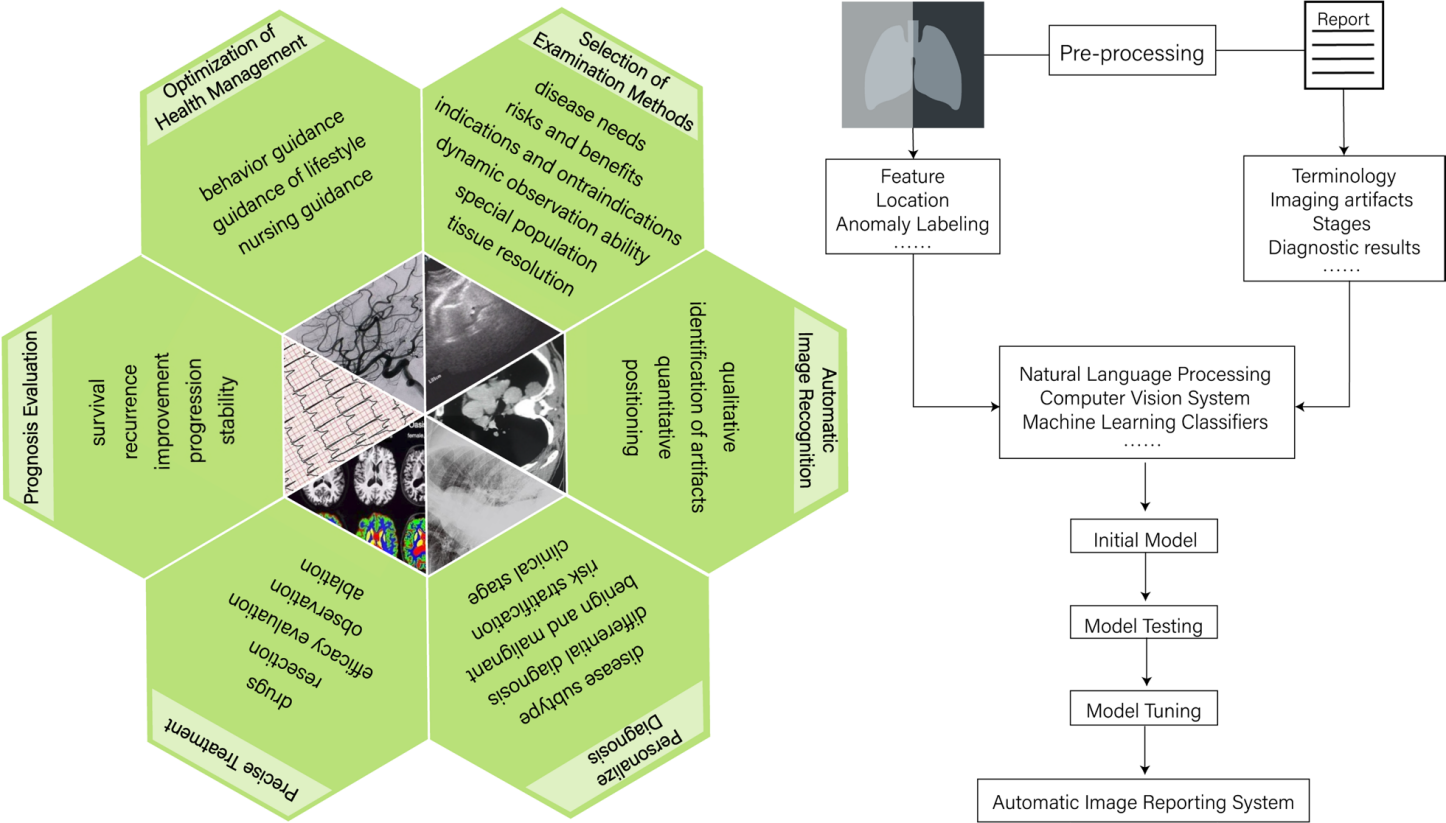
Combination of artificial intelligence and imaging. The combination of artificial intelligence and imaging can help to select examination methods, realize automatic report interpretation, individualized diagnosis, individualized treatment, prognosis evaluation and optimize health management. A case study of artificial intelligence to establish an automatic impact reporting system, which uses a variety of artificial intelligence technologies such as natural language processing and computer vision systems

Clinically, routine VTE screening relies on D-dimer levels, which can result in a high rate of false positives. The gold standard for diagnosing VTE is imaging results, including computed tomography (CT) and ultrasound (US), which can be costly for patients in underdeveloped areas and are also subject to variability due to differences in physician interpretation. By combining natural language processing with machine learning classifiers, VTE can be accurately identified from narrative radiology reports [103]. This approach not only saves clinicians' time in interpreting reports but also ensures result consistency. Antibody detection in the plasma of patients with antiphospholipid syndrome is complex, and there is noticeable heterogeneity between batches. As thrombosis is a significant clinical manifestation of APS, diagnosing APS using a neural network based on thrombin generation data is possible, and the results demonstrate good sensitivity and specificity [104]. In recent years, computer vision imaging has become increasingly common in assisting image interpretation, with radiomics representing its most prominent application [105]. Whether in coronary angiography or brain magnetic resonance imaging, deep learning (DL) can learn from large datasets to integrate image information from multiple aspects, achieving automated and multimodal thrombosis diagnosis. Two deep neural networks are utilized to learn segment information and lesion morphology in coronary angiography images, respectively [106]. Finally, an automated angiography reporting system is generated, improving the accuracy of angiography diagnosis and enhancing the consistency of treatment decisions. Similarly, DL is applied to the diagnosis of cerebral venous thrombosis by brain magnetic resonance imaging. By analyzing the images of different sequences together, the information complementarity between sequences is realized, and the sensitivity of diagnosis is improved [107] (shown in Fig. 7).

### Other uses

AI holds great potential in several areas of medicine such as clinical decision-making, drug discovery, and medical research. Thrombosis is a condition that can be treated through several methods, including anticoagulation, thrombolysis, interventional therapy, surgical treatment, and supportive therapy. Selecting the appropriate treatment method is vital for clinical outcomes.

Platelet aggregation is the primary mechanism involved in the formation of atherosclerotic thrombosis. Agonists and platelet surface receptors combine to trigger the activation process. While there are antiplatelet drugs available for different types of agonists, there is no clear plan for selecting which drug to use. High-throughput imaging flow cytometry combined with image data analysis can identify subtle differences between platelet aggregates that have been activated by different types of agonists [108]. According to the different mechanism of platelet aggregation, the corresponding antiplatelet drugs can play a more accurate and efficient role. Using artificial neural network to integrate plasma proteomics and genome-wide association analysis data, a new pulmonary embolism susceptibility locus PLXNA4 was identified [109]. This finding provided a new direction for the study of the mechanism of PE.

In recent years, new technologies such as AI and in vivo nanotechnology have been used to predict, diagnose, and treat thrombosis. Nano-scale inflatable bubbles can be used as a drug delivery system that targets local areas, improving the efficacy while avoiding the toxic effects caused by off-target. Contrast agents can also be loaded onto the bubbles to make molecular imaging possible. When combined with AI, this approach can guide nanobubbles to improve the safety and real-time performance of precision medicine [110]. Microbubbles are a promising tool for the treatment of thrombotic diseases. They can open blocked blood vessels, target the delivery of thrombolytic drugs, and minimize the risk of adverse reactions such as bleeding. Tumor-derived extracellular vesicles (EVs) in cancer patients contain procoagulant components that can lead to venous thromboembolism (VTE). To detect VTE risk more accurately and specifically, a procoagulant extracellular vesicle barcode composed of TiO2 nanoflowers (TiNFs) can be used for in-situ detection [111]. This approach can be coupled with machine learning-assisted data analysis for improved accuracy and stability. The test results are not affected by the instantaneous hypercoagulable state of the blood since tumor-derived EVs continue to secrete.

Medical artificial intelligence (AI) holds great potential for improving clinical outcomes, but there are still many challenges to overcome before it can be widely implemented. First of all, from the data set level, there are data barriers between hospitals. Different units may have heterogeneity in examination results and disease interpretation, making it challenging to develop models that are universally applicable. While the medical system has shifted towards electronic data management, the integrity, consistency, and accuracy of data still need to be improved to ensure high-quality modeling. The cost of large-scale multi-center research poses another challenge, limiting the development of many prediction models to single institutions. The sample size of training sets, test sets, and verification sets is often not large enough, leading to biased parameters. External validation of patient data from other institutions is required to make the model more universal. This validation process can also help identify and correct any biases or inaccuracies in the original model. Secondly, from an algorithmic standpoint, as artificial intelligence advances from binary classifiers to multiclass classifiers, the demand for computational power increases, necessitating more sophisticated technical support. It can be time-consuming to determine the ML model with the best performance. The calculation work and a large number of adjustments to the hyperparameters depend on personnel with professional knowledge, which can create a gap in talent. Automated machine learning may be more practical for future clinical medical research to address these issues. Finally, from the perspective of the operation mode of artificial intelligence, the widespread adoption of medical artificial intelligence faces many challenges. Firstly, establishing effective clinical models requires ample support from medical big data, inevitably bringing risks of patient privacy breaches. Additionally, many AI models, especially those based on neural networks, struggle with algorithmic opacity, often making it difficult to clearly explain their decision-making processes. This lack of transparency can diminish patient trust in the models, thereby hindering the broad application of medical AI technologies. Ethical and moral considerations are also crucial when using AI for medical decisions. Given that AI learns from clinical data, which may reflect human biases and treatment preferences, ensuring fairness in the model's application is likewise a significant challenge.

Overall, there is still much room for development in medical AI. To achieve real clinical transformation, data barriers between hospitals must be addressed, sample sizes for modeling must be expanded and validated externally, and automated machine learning should be prioritized as a more efficient and universal approach. By identifying and addressing these challenges, we can unlock the full potential of medical AI to improve patient outcomes and healthcare overall.

## Optimizing thrombotic risk assessment: constructing the ideal risk scoring table

Thrombosis is a common yet serious clinical issue, with significant implications for the prevention and management of thromboembolic events. Existing thrombotic risk assessment scales play a crucial role in clinical practice; however, they face various limitations such as inadequate model universality and inconsistent predictive accuracy. Therefore, there is a recognized need to develop scoring systems with higher sensitivity and accuracy to enhance predictive capabilities for thrombotic risk and improve clinical applicability.

In recent years, there have been attempts to utilize artificial intelligence (AI) in constructing thrombotic risk assessment scales [112–114]. By employing suitable algorithms in the construction process, researchers have successfully developed novel thrombosis prediction models that may hold clinical significance. For instance, Xi et al. utilized eight machine learning algorithms including Random Forest (RF) in a retrospective study of clinical data to identify RF as having optimal diagnostic performance for reducing misdiagnosis and missed diagnoses of acute pulmonary embolism (APE) [112]. Variables included in their model were D-dimer, cardiac troponin T (cTNT), arterial oxygen saturation, heart rate, chest pain, leg pain, hemoptysis, and chronic heart failure. Subsequently, the RF model, alongside established Wells and revised Geneva scores and the Years algorithm, predicted outcomes in a test set, demonstrating superior performance compared to the Wells score and comparable diagnostic accuracy to other assessment strategies. This suggests that AI can assist researchers in refining and selecting critical clinical features, eliminating redundant information, and enhancing the efficiency of clinical decision-making. Another study the same year utilized structured electronic health records (EHR) for learning, constructing a model for VTE diagnosis and one-year risk prediction [113]. This model can make real-time predictions based on changes in patient clinical data. Such a dynamic assessment method aids clinicians in promptly adjusting treatment measures, achieving personalized and precise thrombosis prevention. However, this model's data originated from one hospital cohort and two national biobanks, potentially introducing composition biases in the cohort, such as higher incidence rates of cancer and other underlying diseases compared to the general population, which may affect the weighting of these variables. Furthermore, due to the limitations of retrospective studies, these models only predict thrombosis diagnosis rather than occurrence.

Based on the study of AI methods and thrombotic scoring scales, we propose the following concepts for an ideal risk scoring table: (1) the study should be prospective, avoiding significant biases in the composition of the study cohort related to diseases, ethnicities, regions, etc., ensuring the model's universality. Patients from multicenter and multinational sources constitute the most ideal study cohort; (2) emphasis should be placed on integrating various thrombosis-related information, primarily including age, baseline conditions, medical history, recent conditions, and inpatient status. Scientific methods should be employed for data collection, transformation, and screening to ensure data integrity and quality. For example, automated extraction of patient clinical data from EHR systems and analysis using standardized methods like PCA for dimensionality reduction to obtain detailed and accurate information; (3) during the initial construction phase, multiple prediction models such as logistic regression, decision trees, support vector machines, and neural networks should be used. Models should be evaluated based on metrics such as accuracy, area under the ROC curve, sensitivity, specificity, etc., selecting the model with the best predictive performance for parameter adjustment and optimization; (4) we believe the model should possess dynamic updating capabilities to monitor and reflect changes in patient health status in real time. This real-time feedback capability assists healthcare providers in promptly adjusting prevention and treatment strategies, achieving personalized medical management; (5) in practical clinical applications, the model should be easy to operate and interpret, enabling healthcare providers to make rapid and accurate decisions based on scoring results. By enhancing thromboembolic risk assessment and early detection, we aim to provide more precise and effective tools to support personalized thrombotic risk management, ultimately reducing the incidence and mortality associated with this condition and lowering the burden on healthcare systems.

## Summary

Through excessive coagulation system activation, deficiencies in endogenous anticoagulants, inhibition of the fibrinolytic system, and other mechanisms, hereditary factors play a crucial role in promoting the development of thrombotic diseases. Novel mutation sites have been identified. Frequent susceptibility factors comprise prothrombin G20210A, FVL, protein C and protein S deficiencies, antithrombin SERPINC1, PAI-1 4G/5G polymorphism. Currently, there is no standardized approach for screening procoagulant genes. Clinically, patients with suspected thrombophilia are given the option to choose whether or not to be screened, followed by systematic testing for those with positive initial screening results. This includes laboratory testing and genetic defect screening, involving both antigen and functional level assessments. When testing, appropriate molecular forms should be selected based on the different forms of coagulation molecules in plasma. There is variation in genetic defects among different ethnicities. It is worth noting that patients with coagulation factor V Leiden and/or prothrombin mutation experienced a decrease in the incidence of bleeding-related adverse events during subsequent long-term anticoagulant therapy [115]. However, the ideal scenario is to identify high-risk patients through a simple and efficient risk prediction model, enabling early detection and intervention to prevent thrombosis.

In a multicenter observational cross-sectional study conducted in Brazil, involving 589 surgical patients and 865 clinical patients, Deheinzelin et al. [116] assessed the risk of VTE formation in patients using three models: the American College of Chest Physicians (ACCP) guidelines, the Caprini score, and the International Union of Cardiovascular Consensus Statements (IUAS). They then compared the results with the actual clinical preventive treatment and discovered a discrepancy between the patient's risk classification and the treatment they received. Medical practices are influenced by local medical conditions and the subjective factors of doctors and patients. Ensuring appropriate and rational anticoagulant therapy remains a clinical challenge. While rating scales continue to improve, manual assessment still constrains the scalability of the scale. Moreover, subjective differences in disease assessment among doctors make it challenging to ensure consistency in diagnosis and treatment approaches. Therefore, artificial intelligence holds great potential for development in the medical field. Currently, AI has achieved significant breakthroughs in risk assessment, auxiliary diagnosis, and treatment guidance. In the future, there is also a wide range of opportunities for further development in areas such as big data mining, follow-up management, and medical research platforms. However, during the development of clinical models, attention should be paid to the phenomenon of overfitting, which can lead to excessive sensitivity.

Currently known thrombotic diseases involve multiple genetic factors. However, due to the fact that most mutations occur only within individuals, their clinical significance remains somewhat limited. Future research directions could focus on how to apply these discoveries in clinical practice. For example, certain mutations that promote thrombosis could be explored for their potential applications in treating bleeding disorders. Currently, treatment of thrombotic diseases primarily relies on symptomatic therapies such as anticoagulants and thrombolytics. Nevertheless, considering their propensity for recurrence, gene therapies like CRISPR-Cas9 gene editing may offer new avenues to fundamentally alleviate or even cure thrombotic conditions. Yet, how researchers can identify potential research subjects from vast datasets remains a significant challenge. In the future, researchers may leverage the power of artificial intelligence to pinpoint suitable research sites through medical research platforms. With advancements in AI technology, clinical diagnosis and treatment will become more personalized and precise, significantly enhancing safety and efficiency. However, transparency in medical decision-making and the protection of patient privacy continue to pose major challenges limiting the widespread application of medical AI. Nevertheless, we believe these will no longer be issues in the near future.

## Data Availability

No datasets were generated or analysed during the current study.
